# Effectiveness of noncontrast abdominal multidetector CT for evaluating the patient with renal insufficiency in the emergency department

**DOI:** 10.1186/cc13268

**Published:** 2014-03-17

**Authors:** HJ Lee, E Kang, JH Shin

**Affiliations:** 1 SMG-SNU Boramae Medical Center, Seoul, South Korea

## Introduction

Contrast-enhanced abdominal multidetector CT (MDCT) is an important and accurate diagnostic approach for acute abdominal symptoms or searching for an infection focus such as sepsis. But contrast dye can cause renal damage especially in critically ill patients. Noncontrast CT can be alternative options but few studies have shown the effectiveness of noncontrast MDCT. We want to evaluate the effectiveness of noncontrast abdominal MDCT in the emergency department.

## Methods

This is a retrospective chart review study conducted in a single tertiary academic hospital from January 2011 to April 2012. Patients were enrolled if they visited the emergency department with acute abdominal symptoms or infection signs, had elevated serum creatinine level, and received noncontrast 16-channel MDCT.

## Results

During study period, 78 patients with renal insufficiency received noncontrast abdominal CT. Causes of CT were infection focus (48, 61.5%), abdominal pain (21, 26.9%), GI bleeding (4, 5.2%), abnormal labs (3, 3.9%), ureter stone (1, 1.3%) and abdominal distension evaluation (1, 1.3%). Any abnormal CT findings were detected in 61 (78.2%) patients. For 42 (53.8%) patients, noncontrast CT findings showed diagnostic abnormal findings. For excluding abdominal pathology, 35 (44.9%) were helpful. In one (1.6%) case with hematochezia, noncontrast CT showed no benefit for patient diagnosis. Additional contrast-enhanced CTs were performed in 32 (41%) and additional findings were found in five cases (6.4%). There were 40 patients with severe sepsis or septic shock. Abnormal CT findings were found in 30 (75%) cases. Additional contrast enhanced CT was done for 13 (32.5%) patients but no additional information was detected.

## Conclusion

Performing noncontrast abdominal MDCT to the patient with renal insufficiency for evaluating acute abdominal symptoms, severe sepsis or septic shock in the emergency department was feasible and effective.

